# A Rare Co-Segregation-Mutation in the Insulin Receptor Substrate 1 Gene in One Chinese Family with Ankylosing Spondylitis

**DOI:** 10.1371/journal.pone.0126348

**Published:** 2015-05-15

**Authors:** Ju Rong, Qiuxia Li, Pingping Zhang, Xinyu Wu, Jinxian Huang, Chao Li, Zetao Liao, Yingying Xie, Qing Lv, Qiujing Wei, Tianwang Li, Jianlin Huang, Shuangyan Cao, Yan Shen, Jieruo Gu

**Affiliations:** 1 Division of Rheumatology, the Third Affiliated Hospital of Sun Yat-sen University, Tianhe Road 600, Guangzhou 510630, China; 2 The Institute of Basic Medical Sciences, Chinese Academy of Medical Sciences & Peking Union Medical College and Chinese National Human Genome Research Center, Beijing, China; Penn State University, UNITED STATES

## Abstract

Ankylosing spondylitis (AS; MIM 106300) is a common rheumatic disease with strong genetic components affecting approximately 0.3% of the population. The exact genetic mechanism of AS remains elusive. Our previous study showed that AS could be transmitted in an autosomal dominant inheritance mode and a 6-cM candidate region located on the chromosome 2q36.1-36.3 was mapped in a Chinese family. Mutation screening was conducted within the candidate region in the family and other AS by sequencing, and the novel mutation will be further validated in other AS families, sporadic cases and healthy controls by mass spectrometry. We identified a rare non-synonymous mutation (Arg580Gly) in insulin receptor substrate 1 (IRS1) co-segregated with disease phenotype in patients of the family, which was not found in other AS families, sporadic patients and healthy controls. In the study, we found a rare non-synonymous mutation in IRS1 co-segregation in one Chinese family with AS, which indicated a new candidate disease causative gene for AS.

## Introduction

Ankylosing spondylitis (AS; MIM 106300) is a genetically heterozygous disease characterized by inflammation, new bone formation and ankylosis of sacroiliac joints, hip and spine. In China, the pooled prevalence of AS is around 0.20% to 0.40% [1], which is similar to that in other countries of the world [2]. Over 80% of AS affects young adults and the disability rate after 5 years of symptoms onset reaches 40%-60% [3]. Unfortunately, so far the classified criteria for early diagnosis of AS have not been well established yet [4] and definitely effective treatment available for preventing bone destruction and controlling ankylosis in AS patients is still under investigation.

AS is regarded as a multi-genic disease which hypothetically involves 3–9 genes as estimated in the previous genetic studies [5]. Although human leucocyte antigen B27 (HLA-B*27) has been known as the genetic factor for decades, the clinical fact that only 1–5% of those HLA-B*27 positive individuals develops AS seems unexplainable [6], and HLA-B*27 can account for no more than 30% of the overall genetic risks of this condition [5]. Furthermore, the mechanistic hypotheses of HLA-B*27 causing AS are not well established [7].

Until now, 45 genetic signals have been identified independently associated with AS [8]. HLA-B*27 was found a strong association with AS. Among them, anthrax toxin receptor 2 (ANTXR2), caspase recruitment domain family, member 9 (CARD9), endoplasmic reticulum aminopeptidase 1 (ERAP1), interleukin 12b (IL12B), interleukin 23 receptor (IL23R), kinesin family member 21B (KIF21B), prostaglandin E receptor 4 (PTGER4), runt-related transcription factor 3 (RUNX3), TBK1 binding protein 1 (TBKBP1), tumor necrosis factor receptor superfamily, member 1A (TNFRSF1A), as well as chromosomes 2p15 and 21q22 were identified in Europeans [8, 9]. Two loci between hyaluronan and proteoglycan link protein 1 (HAPLN1)—EGF-like repeats and discoidin I-like domains 3 (EDIL3) and within anoctamin 6 (ANO6) have recently been reported in Han Chinese [10]. In addition to the AS associated loci, 12 AS associated haplotypes have also been identified [8].

Our previous study in Chinese families demonstrated that the disease could be transmitted in an autosomal dominant inheritance as one of the genetic models. Moreover, we originally identified one Chinese family (Family A) candidate region encompassing susceptibility locus to AS in chromosome 2q36 [11]. In this study, extended mutation screening was conducted in the region in the family and sporadic AS patients. We identified a rare co-segregation mutation in the insulin receptor substrate 1 gene (IRS1) in AS patients of Family A through mutation screening, which indicated this new candidate gene to the AS.

## Materials and Methods

### Study Subjects

**Fig 1:**
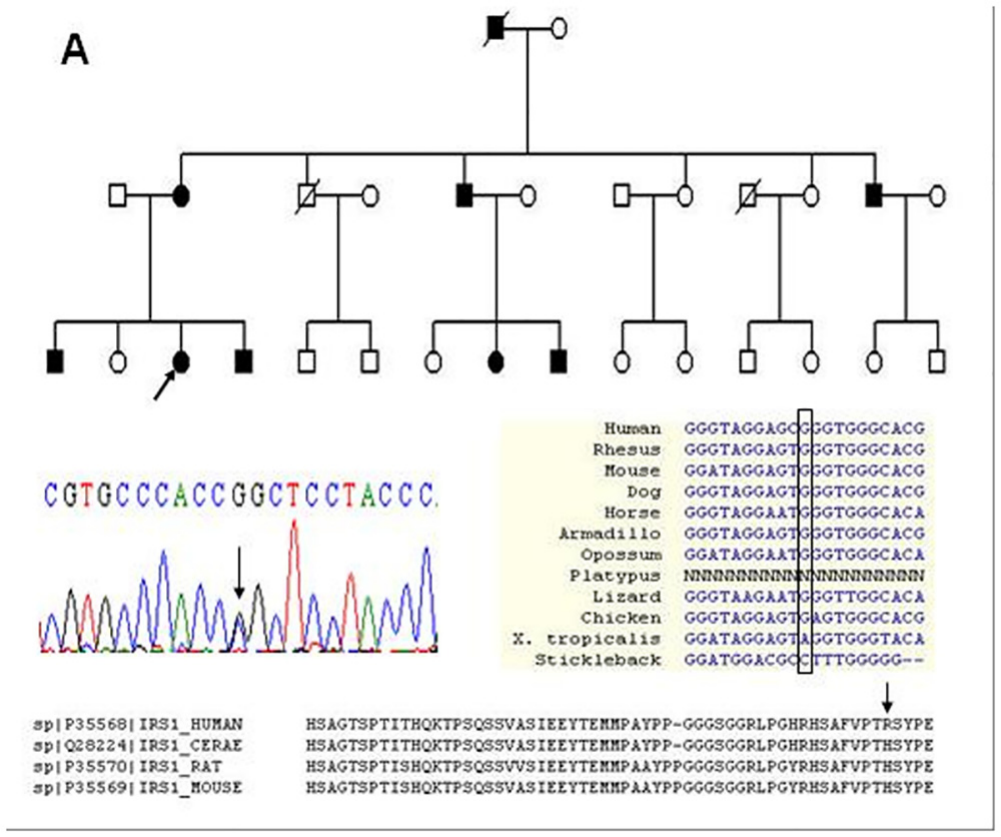
Multiple alignments of non-synonymous heterogeneous variants in IRS1 cosegregated with disease in Family A. The co-segregated variant (1780 C > G transition) in Family A. In this panel, the top graph displays the pedigree structure of Chinese family; the left graph in the middle displays sequencing result of mutated variant; the right graph in the middle displays nucleic acid sequences alignment in multiple species (UCSC Genome Browser); the bottom graph displays amino acid sequences alignment in multiple species (Clustal W).

Five samples including a proband from Family A and two probands from other pedigrees with AS, as well as one patient and one health control from Family A were performed direct sequencing in first stage (Fig 1). 519 ethnically matched subjects contain 210 healthy volunteers, 96 patients with family history of AS and 203 sporadic AS patients were recruited in the second stage and sequenced via mass spectrometry. All the pedigrees originated from the Chinese Han ethnic group. Cases and controls were matched by sex and age (Table 1). The diagnosis of AS was conducted according to the modified New York criteria of AS published in 1984 [10]. The patients with AS were assessed by at least two qualified rheumatologists from our department. Written informed consents were obtained from all the subjects and our study was approved by the ethics committee of the third affiliated hospital of Sun Yat-Sen university.

**Table 1:** The summary of samples analyzed at the second stage.

	AS	Control		P
Male (n)	238	160	X^2^ = 2.861	0.091
Female (n)	71	50		
Age (y)	28.21±8.72	28.14±7.31	F = 8.381	0.098

### Mutation Screening by Direct Sequencing

The promoter region (around 3000 bp upstream of the first exon), exonic region and exon-intron junctions of 30 genes in the candidate region were sequenced. The source of the primers was the NCBI database Results were analyzed with the Phred/Phrap/Consed programme and the polyphred and BioEdit software. Primers were designed using Primer3 or Primer Premier 5.0 and evaluated with Oligo 6. Polymerase chain reaction (PCR) was initiated at 94°C for 5 minutes, followed by 30 cycles of incubation at 94°C for 30 seconds, at an annealing temperature for 30 seconds, and at 72°C for 1 minute. The final step took place at 72°C for 7 minutes. All PCR products underwent purification before direct sequencing with the ABI 3700 sequencer.

### Genotyping

Genotyping was performed in 384-well plate format on the MassARRAY platform (Sequenom, CA, USA). Genomic positions of the genetic variants were selected and 100 bp up- and downstream sequences were used for primer design. The multiplex PCR primers and extended primers for candidate SNPs selected from the previous study were designed by Sequenom MassARRAY Assay Design software version 4.0 (Sequenom, CA, USA) with default parameters (Table 2). PCR amplification were performed in a total volume of 5 μl with 20–50 ng of genomic DNA, 10 mM MgCl_2_, 1 units of PCR polymerase, 2.5 mM dNTP, and 0.5 μM primers. The PCR amplification started at 95°C for 2 min, followed by 45 cycles of 95°C for 30 s, 56°C for 30 s, and 72°C for 1 min, with final extension of 72°C for 5 min. After dephosphorylation of PCR products, PCR products were input to the allele-specific single base primer extension reactions. The extension mixture consisted of 0.200μl 10× iPLEX buffer, 0.200 μl iPLEX termination mix, 0.041 μl iPLEX enzyme (iPLEX Gold Reaction Kit; Sequenom) and 0.940 μl extension primers in a total 9 μl volume. The extension reaction involved preliminary denaturation at 94°C for 30 s, followed by 40 cycles of denaturation at 94°C for 5 s, annealing at 52°C for 5 s, and extension at 80°C for 5 s. The annealing and extension cycle was repeated one more time in a cycle. After allele-specific single base primer extension reactions, polymorphic sites were determined by the matrix-assisted laser desorption/ ionization time-of-flight mass spectrometry (MALDI-TOF MS, SpectroREADER, Sequenom). Resulting genotype data was collected by MassArray Typer software version 4.0 (Sequenom, CA, USA).

**Table 2:** Primers for IRS1 allele genotyping.

IRS1 SNPs	P1	P2	P3
2:227660285	ACGTTGGATGATGCCCCCAGGTCCTTGTG	ACGTTGGATGATCCTCAGCAGCCTCTGCTT	AGCTGGCTGCCCACT
rs144912682	ACGTTGGATGTGCATTTCCAGACCCTCCTC	ACGTTGGATGACACAGGCACTCCGCCTTC	CCGCCTTCGTGCCCACC
2:227600359	ACGTTGGATGGCTTAAAAGAGTAGAATAGGG	ACGTTGGATGGAACCATCTATGGCACTATG	TGTTTTCTGAATTACAATCTTAAAA

### Statistical Analysis

The Hardy—Weinberg equilibrium was assessed by an exact test. The differences between the patients and control groups in genotype and allele frequency was performed using chi-square test or Fisher’s test. All analyses were done by using PLINK software.

### Alignment of Mutated Sites in Multiple Species

The mutated sites of non-synonymous variants were aligned by UCSC Genome Browser (http://genome.ucsc.edu/) in multiple species. Vertebrate Multiz Alignment & PhastCons conservation displayed the density of evolutionary conservation. Meanwhile, the corresponding amino acid sites were aligned by Clustal W in multiple species.

## Results

### Clinical Data

The proband of Family A was a 24-year-old woman. There were 8 affected individuals (male: female = 5:3), 13 genealogical and 5 non-genealogical individuals in the family (Fig 1). Blood specimens were obtained from all the members of Family A. None of affected individuals had a history of ophthalmia, urethritis and cervicitis. Sporadic AS patients had primarily sacroiliac joints and spine involvement.

### Discovery of IRS1 with Novel Variants through Mutation Screening

Mutation screening was conducted within the region of 2q36. A cosegregated variant was identified in the IRS1 gene (GenBank accession number NM_005544) in Family A. A novel 1780 position C to G transition (G1780C) in coding region of IRS1 that led to alteration of arginine to glycine at amino acid position 580 (Arg580Gly) was identified in all 8 available AS patients from Family A. Meantime, two others novel variants were found in the other probands at the first stage, which located in the IRS1 non-coding region, 3’ UTR or promoter region respectively (Table 3).

**Table 3:** Single nucleotide polymorphisms of IRS1 for allele genotyping.

rs ID	Chr	Position^a^	Alleles	Function	AminoAcids
			(reference allele)		
2:227600359	2	227600359	T/C	UTR-3	
2:227660285	2	227660285	A/G	Missense	SER→LEU
rs144912682^a^	2	227661717	C/G	Missense	ARG→GLY

^a^, NCBI Build 37.1 human genome coordinates

### Conservation of Nonsynonymous Variants in the IRS1 Gene

In the Family A, the co-segregated non-synonymous variants G1780C in IRS1 was located in the conserved region of IRS1 as estimated by Mammal Cons on UCSC Genome Browser. The corresponding amino acid was conserved in multiple species as estimated by Clustal W (Fig 1).

### The Result of Variants in the Verification Phase

Allele and genotype frequencies of SNPs were shown in the Table 4 and Table 5. Cases/controls association analysis showed no differences between AS in Chinese Han and matched healthy controls. In the second verification process, the G1780C variant of IRS1 was only detected in all 8 affected members of family A. However, none of sporadic cases and controls had the C allele in 1780 position at the validation stage, it was a G to A transition at the 1780 position.

**Table 4:** Association results for SNPs of IRS1 in samples.

rsID	Position	Alleles (risk/non-risk allele)	Risk allele frequency (case/control)	P value	OR 95%CI
2:227600359	227600359	T/C	0/0.002415	0.4099	NA
2:227660285	227660285	A/G	0.005357/0.005181	0.07221	1.034 (0.9153–0.172)
rs144912682	227661717	G/A^a^	0.98556/0.6701	0.07221	2.329 (0.956–5.673)

^a^, at the sequencing stage,

The single nucleotide polymorphisms of rs144912682 was G/C, but at the validate stage, it was G/A not G/C.

**Table 5:** Genotype for SNPs of IRS1 in samples.

rsID	Position	Genotype	Genotype counts and frequency	
			Case	Control
2:227600359	227600359	TT	0 (0%)	0 (0%)
		TC	0 (0%)	1 (0.5%)
		CC	298 (100%)	206 (99.5%)
2:227660285	227660285	AA	0 (0%)	0 (0%)
		AG	3 (1.1%)	2 (1.0%)
		GG	277 (98.9%)	191 (99%)
rs144912682	227661717	GG	271 (97.8%)	190 (96.4%)
		GA	4 (1.4%)	1 (0.5%)
		AA	2 (0.8%)	6 (3.1%)

## Discussion

AS has been known to be a strongly familial, heritable disease for long periods. Accordingly, considerable effort has been focused on screening and identification susceptibility genes that contribute to this genetic predisposition to AS. To date, a large body of results suggests that HLA region maybe contributed to the pathogenesis of AS, especial HLA-B*27 [8–10]. At the same time, however, convincing evidence has been accumulated for the existence of further non-B*27 MHC genes association with AS over the past decade [8–10]. Recently, we described an autosomal dominant mode in three pedigrees, and found the new candidate region associated with AS located in 2q36.1–2q36.3 in one AS family [11]. This means that the stronger non-HLA-linked genes are also likely to be conferred by the region.

In the current study, we have found that IRS1 may be a disease causative gene for AS patients inherited in an autosomal dominant pattern, and we detected three novel variants of IRS1 in the AS patients. Among the three variants, two polymorphisms (227600359 and 227660285) in the IRS1 have not been reported previously, but the variant occurring in the 1780 position has been characterized (rs144912682). It is worth noting, however, that the nucleotide C change to G in the 1780 position in our study is different from the previously rs144912682 variant that nucleotide C is converted to T. Moreover, the G1780C variant of IRS1 was the finding of co-segregated variant in Family A [11] and did not be found the mutation in other AS patients and controls in this study. The results suggested that the G1780C variant of IRS1 may be new locus susceptible to AS. Of noted the fact that other known susceptibility genes are not located in the 2q36 region, and we did not found linkage between the afore mentioned known genes or loci with those pedigrees in the previous study [11], we do not specifically analyze the status of other known susceptible genes in the Family A members in this study. Additionally, we also have not found the relationship between the IRS1 G1780C with HLA-B*27 in genetic.

IRS-1 encoded by the IRS1 gene, is a primary substrate of the insulin receptor/ insulin-like growth factor receptor (IGF-1R) and functions as a docking protein in cytoplasm. Multiple tyrosine residues within YXXM motif of IRS-1 become rapidly phosphorylated after insulin/ IGF-1 stimulation. Tyrosine phosphorylation of IRS-1 can recruit those proteins containing Src homology-2 domains, including phosphatidylinositol 3-kinase (PI 3-K) [12], growth factor receptor-bound protein 2 (Grb-2) [13], Src homology-2-containing protein tyrosine phosphatase-2 (SHP-2) [14], Fyn [15], and Nck [16], as well as activate a variety of intracellular signaling and generate biological effects. The defects in the pathway are considered to contribute to the pathogenesis of diabetes mellitus (DM). However, functional study of IRS1 in AS has not been developed. Null of IRS1 in mice causes not only insulin-resistant and hyperglycaemia, but also significant growth retardation during intrauterine and smaller size than wild-type (WT) littermates postnatal age in 4 months [17]. Ogata N *et al* reported that IRS-1 is only expressed in osteoblasts and chondrocytes, and no expressed in mature osteoclasts [18], indicating IRS-1 mainly participating in osteoblast activity. Furthermore, osteoblast lacking IRS1 impairs osteoblast proliferation and differentiation, inducing osteoclastogensis, and results in low turnover osteopenia [18]. Meanwhile, homozygous mice lacking IRS1 gene have shown that IRS1 was essential to maintain bone turnover in osteoblast, not only because it is play essential role in insulin/ IGF-1 signaling, but also it may be involved in signal transduction for other related bone metabolic factors such as 1,25(OH)_2_D_3_ [18], adiponectin [19] and parathyroid hormone (PTH) [20]. Thus, IRS-1 signaling might also involve the regulation of cartilage and bone metabolism [21]. Consistent with IRS-1 characteristics in bone formation, we might have found a potential relationship between IRS-1 with new bone formation in AS patients.

In view of the crucial role of IRS-1 in insulin signaling, polymorphisms in the IRS1 gene were considered to have an impact on IRS-1 function and association with diabetes, especially those nonsynonymous mutations. To date, several nonsynonymous mutations in human IRS-1 have been reported, such as Ala512Pro [22], Thr608Arg [23], and Gly972Arg [24, 25], Ser1043Tyr, Cys1095Tyr [26]. Among them, particularly either Thr608Arg or Gly972Arg (rs1801278) variant near YXXM motif was deeply explored. The results shown that the it was mild to moderate impaired the ability of phosphorylated IRS-1 bind to the p85 regulatory subunit of PI 3-K by the variants, and the functional defect of IRS-1 variants play a contributory role in insulin resistance and diabetes susceptibility [23–25]. Not only mutations in exon of IRS1 are associated with diabetes, genetic variants (rs2943641; rs2943650) outside of IRS1 coding region are also though to be responsible for insulin resistance, hyperinsulinemia, dyslipidemia, and DM Type-2 [27, 28]. Despite the detailed experimental data and genetic evidence suggests that these mutations could affect the function of IRS-1, there are not a consistent findings and the effect yet among these studies. The Gly819Arg, the Gly972Arg, and Arg1221Cys variants were also found to be without effect in studies in COS 7 cells [29]. Even if the mutation Met614Val significantly alters the structure of a YXXM motif in IRS1 which is thought to interact with p85 subunit of PI 3-K, no alterations in insulin-stimulated PI3-K activity were found in cells expressing the IRS1 Met614Val variant [30]. In the present study, there are three mutations including two nonsynonymous mutations in IRS1. We have inquired whether the detected amino acid substitutions impair the biologically function of IRS-1. It seems likely that they would affect the translated IRS-1 sequence or influences the functions of IRS-1 by changing the conformation of protein or messenger RNA stability (i.e., the 3’-untranslated region [3’-UTR]). Intriguing, IRS-1 Arg580Gly was especially located in the conservative domain of multiple alignments of different species. However, secondary structures around the three novel variants in IRS-1 have not been resolved; we are unable to predict the nonsynonymous mutations how impact on the function of IRS-1 for lacking structure of homologous proteins to align. Because IRS-1 Arg580Gly is much closer 612–615 YXXM motif than 551–554 YXXM motif, which the 612–615 YXXM motif can bind the p85 subunit after insulin/IGF-1 stimulation. We hypothesized that IRS-1 Arg580Gly would affect the conformation of 612–615 YXXM motif for transition from hydrophilic arginine to hydrophobic glycine, following impairing PI 3-K-dependent signaling pathway in bone metabolism.

In a conclusion, our data first indicated that IRS1 might be a novel disease causative gene for AS. We assume that IRS1 Arg580Gly triggers abnormal bone metabolism in AS patients. It is worthy to further explore the function of the mutation in AS.
